# DNA methylation of genes involved in lipid metabolism drives adiponectin levels and metabolic disease

**DOI:** 10.1007/s00125-025-06549-6

**Published:** 2025-10-08

**Authors:** Lucy Sinke, Thomas Delerue, Rory Wilson, Xueling Lu, Yujing Xia, Ricardo Costeira, M. Kamal Nasr, Marian Beekman, Lude Franke, Alexandra Zhernakova, Jingyuan Fu, Christian Gieger, Christian Herder, Wolfgang Koenig, Annette Peters, José M. Ordovas, Marcus Dörr, Hans J. Grabe, Matthias Nauck, Jordana T. Bell, Alexander Teumer, Harold Snieder, Melanie Waldenberger, P. Eline Slagboom, Bastiaan T. Heijmans

**Affiliations:** 1 Molecular Epidemiology, Department of Biomedical Data Sciences, Leiden University Medical Center, Leiden, the Netherlands; 2 Research Unit Molecular Epidemiology, Institute of Epidemiology, Helmholtz Munich, German Research Center for Environmental Health, Neuherberg, Germany; 3 Department of Epidemiology, University of Groningen, University Medical Center Groningen, Groningen, the Netherlands; 4 Laboratory of Environmental Medicine and Developmental Toxicology, Shantou University Medical College, Guangdong, China; 5 Department of Twin Research and Genetic Epidemiology, King’s College London, London, UK; 6 Department of Psychiatry and Psychotherapy, University Medicine Greifswald, Greifswald, Germany; 7 German Center for Cardiovascular Research (DZHK), Partner Site Greifswald, Greifswald, Germany; 8 Department of Genetics, University of Groningen, University Medical Center Groningen, Groningen, the Netherlands; 9 Department of Paediatrics, University of Groningen, University Medical Center Groningen, Groningen, the Netherlands; 10 German Center for Cardiovascular Research (DZHK), Partner Site Munich Heart Alliance, Munich, Germany; 11 Institute for Clinical Diabetology, German Diabetes Center (Deutsches Diabetes-Zentrum/DDZ), Leibniz Center for Diabetes Research at Heinrich-Heine-University Düsseldorf, Düsseldorf, Germany; 12 Department of Endocrinology and Diabetology, Medical Faculty and University Hospital Düsseldorf, Heinrich-Heine-University Düsseldorf, Düsseldorf, Germany; 13 German Center for Diabetes Research (DZD), Partner Düsseldorf, München-Neuherberg, Germany; 14 Technical University of Munich, School of Medicine and Health, German Heart Centre, TUM University Hospital, Munich, Germany; 15 Institute of Epidemiology and Medical Biometry, University of Ulm, Ulm, Germany; 16 Institute for Medical Informatics, Biometrics and Epidemiology, Ludwig-Maximilians-Universität München, Munich, Germany; 17 USDA ARS, Nutrition and Genomics Laboratory, JM-USDA Human Nutrition Research Center on Aging at Tufts University, Boston, MA USA; 18 Department of Internal Medicine B, University Medicine Greifswald, Greifswald, Germany; 19 German Center for Neurodegenerative Diseases (DZNE), Site Rostock/Greifswald, Germany; 20 Institute of Clinical Chemistry and Laboratory Medicine, University Medicine Greifswald, Greifswald, Germany; 21 Institute for Community Medicine, University Medicine Greifswald, Greifswald, Germany

**Keywords:** Adiponectin, Causal inference, Epigenomics, Leptin, Lipid metabolism, Meta-analysis, Metabolic health, Type 2 diabetes

## Abstract

**Aims/hypothesis:**

Despite playing critical roles in the pathophysiology of type 2 diabetes and other metabolic disorders, the molecular mechanisms underlying circulating adipokine levels remain poorly understood. By identifying genomic regions involved in the regulation of adipokine levels and adipokine-mediated disease risk, we can improve our understanding of type 2 diabetes pathogenesis and inter-individual differences in metabolic risk.

**Methods:**

We conducted an epigenome-wide meta-analysis of associations between serum adiponectin (*n*=2791) and leptin (*n*=3661) and leukocyte DNA methylation at over 400,000 CpG sites across five European cohorts. The resulting methylation signatures were followed up using functional genomics, integrative analyses and causal inference methods.

**Results:**

Our findings revealed robust associations with adiponectin at 73 CpGs and leptin at 211 CpGs. Many of the identified sites were also associated with risk factors for the metabolic syndrome and located in enhancers close to relevant transcription factor binding sites. Integrative analyses additionally linked 35 of the adiponectin-associated CpGs to the expression of 46 genes, and 100 of the leptin-associated CpGs to the expression of 151 genes, with implicated genes enriched for lipid transport (e.g. *ABCG1*), metabolism (e.g. *CPT1A*) and biosynthesis (e.g. *DHCR24*). Bidirectional two-sample Mendelian randomisation further identified two specific CpG sites as plausible drivers of both adiponectin levels and metabolic health: one annotated to *ADIPOQ*, the gene encoding adiponectin; and another linked to the expression of *SREBF1*, an established modifier of type 2 diabetes risk known to exert its effects via adiponectin.

**Conclusions/interpretation:**

Taken together, these large-scale and integrative analyses uncovered links between adipokines and widespread, yet functionally specific, differences in regulation of genes with a central role in type 2 diabetes and its risk factors.

**Graphical Abstract:**

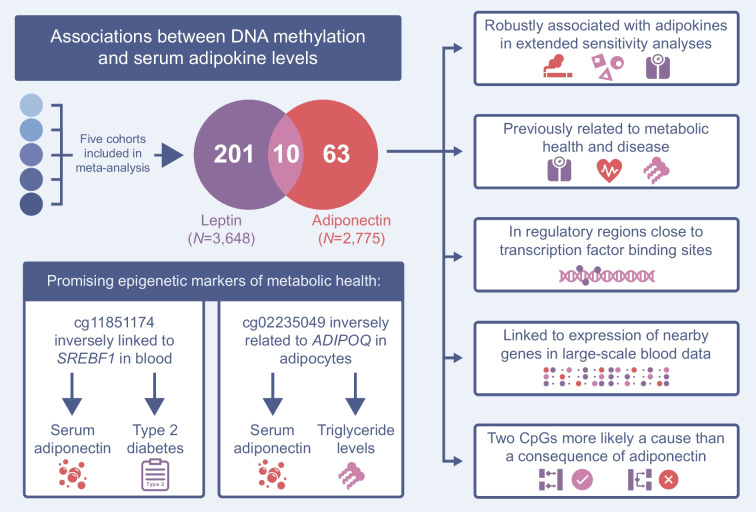

**Supplementary Information:**

The online version contains peer-reviewed but unedited supplementary material available at 10.1007/s00125-025-06549-6.


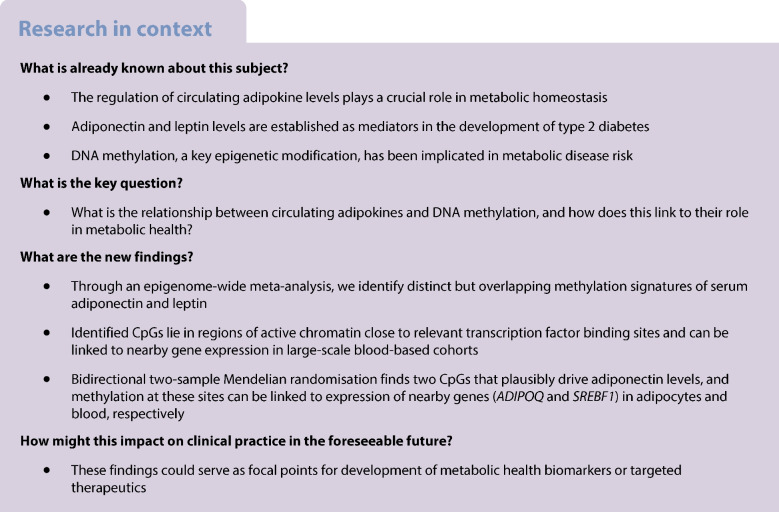


## Introduction

Adiponectin and leptin are key adipokines that play central roles in regulating energy homeostasis and metabolic processes, with influences on insulin sensitivity and inflammation. Circulating levels of these adipokines are directly implicated in the pathogenesis and progression of the metabolic syndrome and type 2 diabetes [1, 2], and a clearer understanding of their regulation could uncover new avenues for predicting, preventing or treating metabolic disease.

Epigenetic modifications, such as DNA methylation (DNAm), are established as being both responsive to lifestyle changes and capable of modifying disease risk. Growing evidence supports epigenetic regulation of adiponectin and leptin as partly driving inter-individual differences in metabolic health [3, 4]. Blood-based epigenome-wide association studies (EWAS) have uncovered robust and biologically meaningful correlations between DNAm, metabolic diseases and their risk factors, even where investigated traits are non-haematopoietic in origin [5, 6]. Supported explanations for detected associations in leukocytes include shared upstream drivers such as diet [7], DNAm responses to circulating metabolic traits [8] and immune cell mediation of the inflammatory components of metabolic disease [9].

Despite substantial progress, however, research directly examining relationships between adiponectin and DNAm have been limited in sample size [10], and leptin has thus far not been investigated on a genome-wide scale. A comprehensive EWAS of these adipokines is warranted in a sufficiently large sample size to detect subtle molecular effects, with thorough interpretation of the resulting methylation signatures for these critical metabolic markers.

## Methods

### Cohort analyses

#### Main analysis

All contacted cohorts with sufficient data followed a common analysis plan (see Cohort Descriptions in electronic supplementary material [ESM] Methods for details), and all samples analysed were taken from distinct individuals (i.e. there were no repeat measurements included in the analysis). DNAm was measured either by the Illumina Infinium HumanMethylation450 (in Leiden Longevity Study [LLS], Cooperative Health Research in the Region of Augsburg [KORA], TwinsUK and LifeLines DEEP [LLD] cohorts) or MethylationEPIC BeadChip array (in Study of Health in Pomerania [SHIP]-TREND cohort). Adipokine measurements below the limit of detection and outlying values for DNAm, adiponectin and leptin (more than three IQRs from the nearest quartile) were removed prior to analysis. Cell-type proportions were predicted from DNAm data using the IDOL algorithm [11].

For each of $j$ CpGs measured in $i$ individuals, a linear regression model (see Equation 1 for general specification) was fitted of DNAm β values on natural log-transformed adiponectin (µg/ml) or leptin (ng/ml). All models were adjusted for age (in years), sex, cell-type proportions predicted from DNAm data (monocytes, CD8^+^ T cells, CD4^+^ T cells, natural killer [NK] cells and B cells) and technical covariates (left to the analyst’s discretion). Sex was considered in the study design and included as a covariate in statistical models to address potential biological differences.

1 $${\text{DNAm}}_{{\beta }_{ij}}= {\upbeta }_{0}+{\upbeta }_{1}\, {{\text{log}}_{\text{e}}\left(\text{adipokine}\right)}_{i}+{\upbeta }_{2} \,{\text{age}}_{i}+ {\upbeta }_{3} \,{\text{sexFemale}}_{i}+{\upbeta }_{4}\, {\text{CD}8\text{T}}_{i}+{\upbeta }_{5} \,{\text{CD}4\text{T}}_{i}+{\upbeta }_{6}\, {\text{NK}}_{i}+{\upbeta }_{7}\, {\text{Mono}}_{i}+{\upbeta }_{8} \,{\text{technical}\_\text{factors}}_{i}$$

Analyses were not stratified by sex, and gender identity was not recorded.

#### Sensitivity analyses

Effects of adjustment for smoking on the relationship between adipokines and our identified CpGs was investigated in a sensitivity analysis, where each cohort added trichotomous smoking to the cohort-specific base models as a categorical fixed effect. In some cases, this resulted in a reduction of the sample size as there was missingness in the smoking data. To distinguish BMI-independent signals, cohorts also ran an additional analysis adjusting for BMI (measured in kg/m^2^).

Sensitivity analyses also investigated the effect of adjusting for extended cell types, estimated using the epiDISH Bioconductor package release 3.20 [12], which became available in the timeframe of this project. Basophils, memory B cells, naive B cells, CD4^+^ memory T cells, CD4^+^ naive T cells, CD8^+^ memory T cells, CD8^+^ naive T cells, eosinophils, monocytes, NK cells and regulatory T cells were added to the base model for all cohorts. Neutrophils were excluded to avoid collinearity as proportions for all cells sum to 1.

### Meta-analysis

Results from each cohort were inspected and rows were removed if they were estimated from fewer than 50 observations. Probes located on sex chromosomes, in ENCODE Blacklist regions [13], or that contained known common genetic variants or were ambiguously mapped [14] were also removed. To ensure good quality data, we inspected QQ, volcano and Manhattan plots, alongside boxplots of the effect size and SE distributions across cohorts. Following these steps, data were available on 412,224 CpGs from the base adiponectin model and 406,832 CpGs for the base leptin model.

The Bioconductor package bacon [15] estimated and adjusted for bias and inflation of the test statistics, using default priors (α=1.28, β=0.36). After running bacon, inflation and bias were estimated at ~1.00 and within ±0.00 for all models, respectively. Bacon-adjusted effect sizes and SEs were used as input in a fixed-effects meta-analysis in METAL version 2011-03-25 [16]. Separate analyses were performed for each of the base models and each extended model (adjusted for smoking, BMI and extended cell counts). Any CpGs for which there was evidence of high heterogeneity in effect sizes between cohorts (*I*^2 ^≥ 80%) would have been removed but there were none. CpGs were regarded as significantly associated with the relevant adipokine if the false discovery rate (FDR)-adjusted *p* value was below 0.05, and only CpGs that still met this criterion in the additional sensitivity analyses for smoking, cell-type proportions and BMI were taken forward into downstream analyses.

### Follow-up analyses

#### Differentially methylated regions

To assess distinct genomic loci associated with circulating adipokine levels, differentially methylated regions (DMRs) were identified using the DMRfinder algorithm [17], as implemented in the DNAmArray workflow version 2.1 [18]. DMRs were defined as regions with at least three differentially methylated positions (DMPs) and an inter-CpG distance of less than 1 kb, allowing a maximum of three non-DMPs across a DMR. The number of distinct loci was calculated as the total number of DMPs minus the number of DMPs in DMRs plus the number of DMRs called by DMRfinder.

#### EWAS enrichment

Using summary data from the EWAS catalogue [19] and EWAS atlas [20], our CpGs were investigated for previous associations with other phenotypes. Any EWAS meeting the following criteria was removed: without an associated PubMed ID; with a sample size under 500; that reported fewer than 100 CpGs in the respective database; missing nominal *p* values; not performed in adults; or not using whole blood or leukocyte samples. Traits were also recoded to ensure consistency between names, for example by combining EWAS of ‘BMI’ and ‘body mass index’. This resulted in a list of 57 traits, which were tested for enrichment of associations with our CpGs using logistic regression.

#### Chromatin state enrichment

Identified CpGs were annotated to chromatin state using the peripheral blood mononuclear cell (PBMC) Roadmap reference epigenomes [21]. Logistic regression models were fitted using the glm function in R to calculate and test ORs for each of the 15 chromatin states. Nominal *p* values were adjusted for multiple testing using FDR and enrichments or depletions were assessed at a 5% significance threshold.

#### Transcription factor binding site enrichment

A 50 bp window around FDR-significant CpGs was scanned using findMotifsGenome.pl from HOMER version 3.1 for enrichment of known motifs compared with a random genomic background matched for GC content [22]. ENCODE transcription factor (TF) binding site (TFBS) annotation for 171 TFs and CpGs on the 450k array was used to further investigate the size of binding sites and distance from CpG to summit [14]. TFs associated with enriched TFBS were examined for links with adipokines and, specifically, adiponectin and leptin pathways and interactions.

#### Integrative analyses

Measurements of blood-based gene expression alongside DNAm from the same samples was available from the Biobank-based Integrative Omics Studies (BIOS) consortium (*n*=3152). This dataset comprises six Dutch biobanks: the Cohort on Diabetes and Atherosclerosis Maastricht [23]; LifeLines [24]; LLS [25]; Netherlands Twin Register [26, 27]; Rotterdam Study [28]; and the Prospective ALS Study Netherlands [29]. After filtering out non-autosomal and lowly expressed genes, count data were transformed into log_2_ counts per million (CPM) using edgeR, and values for each gene were rank inverse normal (RIN)-transformed prior to analysis [30].

Genomic locations of human transcripts, exons, coding sequences and genes were imported from the Ensembl database using makeTxDbFromEnsembl from the GenomicFeatures Bioconductor package [31]. These were used to identify the nearest gene to each adipokine-associated CpG and to save a list of all genes within 100 kb of each CpG. To examine links between DNAm and gene expression, linear regression models were fitted with RIN-transformed log_2_CPM values as the response variable and methylation β values as the independent variable, adjusting for the effects of age, sex, technical covariates (row, plate, and flowcell) and 12 blood-cell counts predicted from DNAm using EpiDISH release 3.20 [12].

For investigations into links between expression and DNAm in Simpson-Golabi-Behmel syndrome (SGBS) pre-adipocytes, publicly available data were downloaded from Gene Expression Omnibus (GEO) using GEOquery in R release 3.20 (https://www.bioconductor.org/packages/release/bioc/html/GEOquery.html). Data were available for the same samples, with expression profiled using the Illumina HumanHT-12 V4.0 expression BeadChip microarray and DNAm profiled using the Illumina Infinium HumanMethylation450 BeadChip array [32]. Count data were normalised to log_2_CPM values and values from probes interrogating *ADIPOQ* (ILMN_1775045) and *SREBF1* (ILMN_1663035, ILMN_1695378 and ILMN_2328986) were extracted. Additionally, β values from cg11851174 and cg02235049 were subset from the DNAm data. Complete information was available for 38 samples across five timepoints (days 0, 1, 2, 4, 8 and 16). Expression and DNAm values were plotted against one another for the relevant comparisons, correlation coefficients were calculated and linear regression models were used for analysis.

#### Over-representation analysis

On the basis of the large-scale blood-based integrative analysis in BIOS, a list of CpGs for which there was evidence for epigenetic regulation of nearby gene expression in leukocytes was saved. The associated gene names were used as input for over-representation analysis using 11 recent (updated in the last 6 years) databases relating to human health and disease downloaded from Enrichr (BioPlanet 2019, Elsevier Pathway Collection, GeDiPNet 2023, GO Biological Process 2023, KEGG Human 2021, MSigDB Hallmark 2020, OMIM, PhenGenI Association 2021, PheWeb 2019, Reactome 2022 and WikiPathway Human 2021). These databases were imported into R and analyses were performed using the Enrichr function from clusterProfiler release 3.20 [33]. *p* values were FDR-adjusted for multiple testing and significance was assessed at the 5% level.

#### Bidirectional two-sample Mendelian randomisation

To assess the direction of effects between adipokines and DNAm at identified CpGs, the TwoSampleMR package was used to perform bidirectional two-sample Mendelian randomisation (2SMR) [34]. This instrumental variable (IV)-based method uses genome-wide association study (GWAS) summary statistics to infer whether a risk factor causally influences an outcome. 2SMR relies on several key assumptions, namely that instruments are relevant, independent and that there is no horizontal pleiotropy. To interrogate the effects of DNAm at our CpGs on adiponectin and leptin, we extracted SNP-based *cis*-methylation quantitative trait locus (mQTL) data from the Genetics of DNA Methylation Consortium (GoDMC) [35] and combined these with summary statistics from recent, large-scale GWAS of both adiponectin [36] and leptin [37]. For some CpGs (42.5% for adiponectin and 38.4% for leptin), there was insufficient data available to interrogate the effects of DNAm at that CpG. For the remaining CpGs, between one and four independent SNPs with data on both their *cis*-association with DNAm and association with the relevant adipokine were used as instruments. These were combined using the Wald ratio (for single mQTL instruments) or inverse variance weighted (IVW) methods (for multiple, independent mQTLs).

To interrogate the influence of adipokine levels on DNAm at identified CpGs, independent GWAS variants from recent, large-scale analyses [36, 37] were used. Of the 18 variants that could instrument adiponectin, there were *trans*-mQTL data in GoDMC available for four of them and, of the six variants that could instrument leptin, there were available data for one. Linkage disequilibrium (LD) proxies with *R*^2^>0.8 for the remaining SNPs were downloaded from the NIH’s LDlink tool version 5.6.7_20240620 [38], and GoDMC data [35] were extracted for these where available. This process identified two other instrumental SNPs that could instrument the adipokines, one for each, meaning that leptin was instrumented by two independent SNPs (rs8043757 and rs4665972) and adiponectin was instrumented by five independent SNPs (rs11023332, rs1108842, rs12051272, rs998584 and rs113086489). The GWAS summary statistics and mQTL effects were then combined using the IVW method and the TwoSampleMR package in R version 0.6.6. For all analyses, *p* values were adjusted for multiple testing using the FDR method and potential causal effects were assessed at the 5% significance threshold.

The following cohorts were used to derive both mQTL effects in GoDMC and adiponectin and/or leptin GWAS effects and therefore had overlapping individuals in both the exposure and outcome datasets for the 2SMR analysis: Rotterdam Study (GoDMC 1472 samples, leptin GWAS 3932 samples); and TwinsUK (GoDMC 843 samples, adiponectin GWAS 968 and 1229 samples, leptin GWAS 5654 samples). Therefore, overall, the overlap was low considering that all three meta-analyses incorporated data from over 16 cohorts.

The TwoSampleMR package version 0.6.6 was also used to interrogate causal links between DNAm at CpGs and metabolic traits. CpGs were instrumented with independent *cis*-mQTLs obtained from GoDMC, and ieugwasr was used to extract MR instruments for the metabolic traits. Reference numbers for the investigated traits were as follows: type 2 diabetes (ebi-a-GCST006967); fasting insulin (ebi-a-GCST9002238); triglycerides (ieu-b-111); HDL-cholesterol (ieu-b-109); and BMI (ieu-b-40).

#### Triangulation analyses

To perform triangulation analyses, we interrogated the correlation between the observed effect of an IV on an outcome (i.e. mQTL–adipokine or polygenic score [PGS]–DNAm associations) and the predicted effect via the exposure. This analysis assumes that if the effect of an exposure on an outcome is causal, it would be possible to predict the IV’s effect on the outcome through a combination of its effect on the exposure and the exposure’s effect on the outcome.

In detail, when looking at the effect of DNAm on adipokine levels (consequential analysis), the ‘observed effect’ is the association between the top mQTL and log_*e*_(adipokine), extracted from the full GWAS summary data. The ‘predicted effect’ combines mQTL and EWAS statistics to estimate the influence of an additional effect allele (EA) on the outcome (i.e. the adipokine). For each additional EA, the expected rise in DNAm at the CpG is equivalent to the mQTL effect size (β_mQTL_). As the EWAS effect size represents the DNAm effect associated with a one-unit increase in the adipokine level, the expected increase in the adipokine level for a β_mQTL_ increase in DNAm can be calculated as the product of the mQTL and EWAS effects (i.e. β_mQTL_ × β_EWAS_). SNP effects on DNAm (mQTL effects) were extracted from GoDMC data [35] and CpG-adipokine effects were extracted from the EWAS meta-analysis presented here.

When looking in the reverse direction (i.e. adipokines as a cause of DNAm), the ‘observed effect’ is a PGS, where the influence of adipokine-associated SNPs on DNAm are weighted by their EA frequency (EAF). The ‘predicted effect’ here uses equivalent EAF weighting and is calculated as PGS~adipokine/adipokine~CpG. The observed and predicted effects in both directions were visualised using scatter plots and correlation was assessed with Pearson correlation coefficients.

### Software

Unless stated otherwise, all calculations were performed using R version 4.2.2 (R Core Team, http://www.r-project.org). For all meta-analyses, METAL, version 2011-03-25 was used (http://csg.sph.umich.edu/abecasis/Metal) [16]. TFBS enrichment analyses were performed using HOMER version 3.1 (http://homer.ucsd.edu/homer) [22].

## Results

### Circulating adipokines have distinct DNA methylation signatures in blood

We performed a meta-analysis of EWAS of circulating adiponectin (*n*=2791; 412,224 CpGs) and leptin (*n*=3661; 406,390 CpGs) levels in blood samples from five European cohorts (Tables 1, 2). Mean age was 55.5 years in the leptin meta-analysis and 56.8 years for adiponectin, and the population was predominantly female (55.2% in the adiponectin meta-analysis, 54.4% in the leptin meta-analysis). Cohorts represented a combination of fasted (KORA, TwinsUK, SHIP-TREND and LLD) and non-fasted (LLS) samples. Full summary statistics for all tested CpGs can be found in ESM Tables 1, 2. Circulating levels of adiponectin and leptin were associated with blood-based DNAm at 73 CpG sites and 621 CpG sites, respectively (*p*_fdr_≤0.05, nominal *p* value thresholds 8.8 × 10^−6^ for adiponectin, 7.6 × 10^−5^ for leptin). These results were adjusted for age, sex, technical covariates and six blood-cell types predicted using DNAm data (granulocytes, monocytes, NK cells, CD4^+^ T cells, CD8^+^ T cells and B cells). No CpGs displayed high heterogeneity between cohorts (all *I*^2^<80%) and test statistics were corrected for bias and inflation.

**Table 1:** Characteristics of cohorts included in the adiponectin EWAS meta-analysis

Characteristic	KORA F4	LLS	LLD	TwinsUK	SHIP-TREND
Sample size	807	718	701	124	441
Age, years	68.8±4.4	58.9±6.7	45.5±13.1	55.1±11.7	50.0±13.4
Female sex	396 (49.1)	370 (51.5)	411 (58.6)	124 (100.0)	241 (54.6)
BMI, kg/m^2^	26.4 (4.2)	25.1 (4.3)	24.7 (4.8)	25.0 (4.7)	27.2 (4.2)
Smoking, current	69 (8.6)	85 (11.8)	130 (18.5)	28 (22.6)	154 (34.9)
Smoking, never	388 (48.1)	199 (27.7)	332 (47.4)	65 (52.4)	173 (39.2)
Adiponectin, µg/ml	9.7 (8.0)	5.3 (3.8)	3.7 (2.7)	7.2 (5.0)	7.0 (5.0)

Data are presented as mean ± SD for age, as median (IQR) for adiponectin and BMI, and as *n* (%) for sex and smoking status

**Table 2:** Characteristics of cohorts included in the leptin EWAS meta-analysis

Characteristic	KORA F4	LLS	LLD	TwinsUK	SHIP-TREND
Sample size	1702	723	701	94	441
Age, years	60.9±8.9	58.9±6.7	45.5±13.1	55.1±11.9	50.0±13.4
Female sex	874 (51.4)	372 (51.4)	411 (58.6)	94 (100.0)	241 (54.6)
BMI, kg/m^2^	27.4 (4.6)	25.1 (4.3)	24.7 (4.8)	25.0 (4.3)	27.2 (4.2)
Smoking, current	247 (14.5)	85 (11.8)	130 (18.5)	12 (12.8)	154 (34.9)
Smoking, never	711 (41.8)	199 (27.5)	332 (47.4)	50 (53.2)	173 (39.2)
Leptin, ng/ml	13.3 (19.9)	12.1 (20.6)	10.0 (17.0)	15.1 (12.1)	10.1 (14.7)

Data are presented as mean ± SD for age, as median (IQR) for leptin and BMI, and as *n* (%) for sex and smoking status

**Fig. 1:**
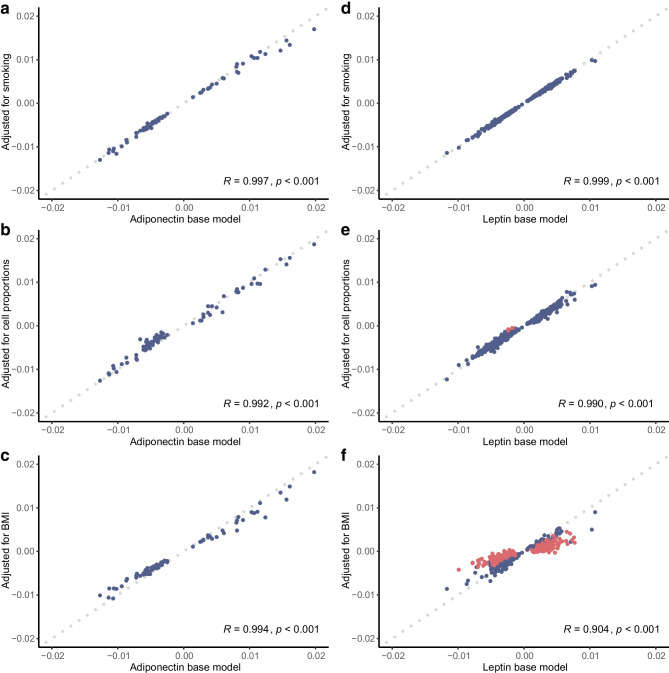
Scatter plots of relationships between adipokine-associated DNAm effects before and after sensitivity analysis with Pearson correlation coefficients and associated *p* values. CpGs taken forward to downstream analysis are shown in blue and removed CpGs are shown in red. A reference line (*y*=*x*) is shown by a grey dotted line in all plots, indicating no change between models. (**a**) Adiponectin-associated effects before and after adjustment for smoking. (**b**) Adiponectin-associated effects before and after adjusting for 12 extended blood-cell-type proportions predicted from DNAm data using EpiDISH. (**c**) Adiponectin-associated effects before and after adjustment for BMI. (**d**) Leptin-associated effects before and after adjustment for smoking. (**e**) Leptin-associated effects before and after adjusting for 12 extended blood-cell-type proportions. (**f**) Leptin-associated effects before and after adjustment for BMI

To evaluate the stability of associations between DNAm and adipokines, sensitivity analyses assessed the impact of smoking, 12 distinct cell types and BMI (ESM Tables 3, 4). For the majority of adiponectin CpGs, associations remained statistically significant after adjustment for these additional variables (*p*_fdr_≤0.05), with very strong correlations between effect size (*R*>0.99, *p*<0.001; Fig. 1a–c). Effects at leptin CpGs also showed relative independence from smoking and cell-type proportions (Fig. 1d, e). Unsurprisingly however, since leptin has stronger and more direct links to obesity [39, 40], 401 leptin CpGs were sensitive to BMI adjustment (*p*_fdr_>0.05; Fig. 1f). To ensure focus on adipokine-specific epigenetic links in downstream analyses, these were removed from the results.

**Fig. 2:**
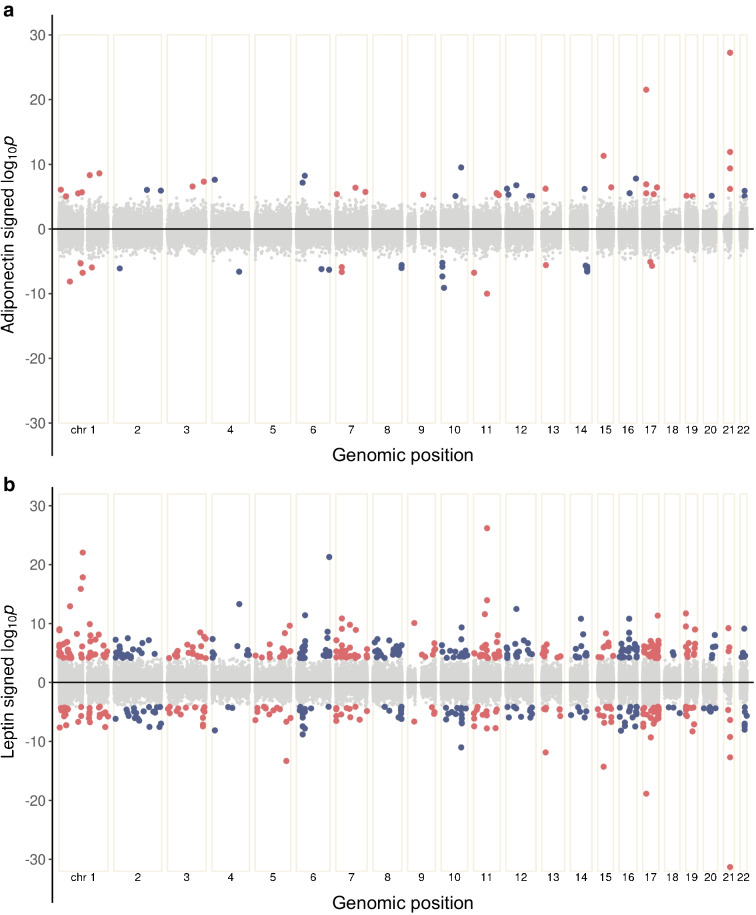
Bidirectional Manhattan plots of the signed log10(*p* values) for all tested CpGs against their cumulative genomic position. Chromosomes are separated by a fixed amount and labelled. CpGs significant at the 5% level after adjusting for multiple testing are shown in red (odd-numbered chromosomes) or blue (even-numbered chromosomes). Non-significant CpGs are shown in grey. (**a**) Results from the adiponectin EWAS meta-analysis. (**b**) Results from the leptin EWAS meta-analysis

**Fig. 3:**
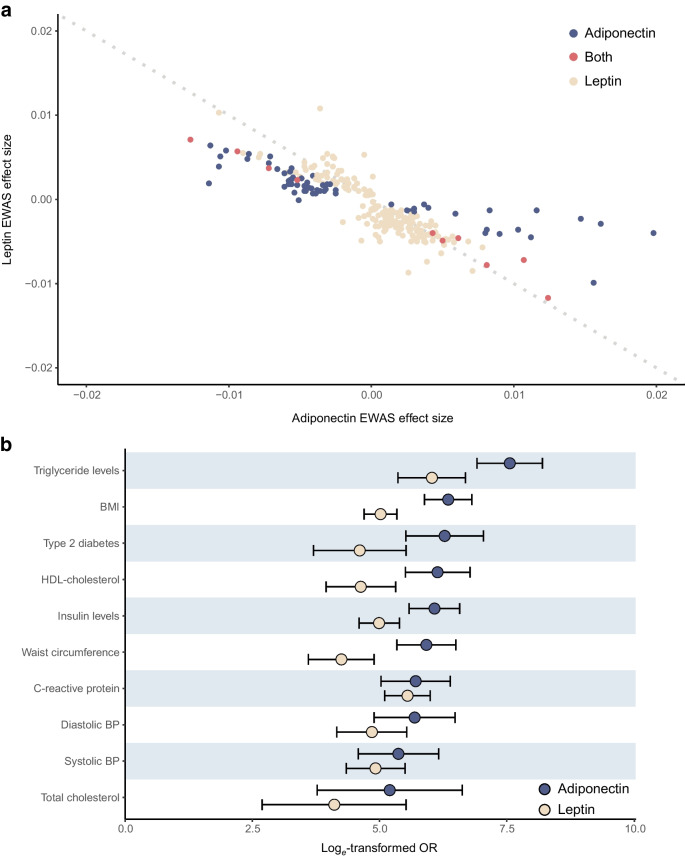
Plots showing the relationships between EWAS of different traits. (**a**) Scatter plot of EWAS meta-analysis effect size for adiponectin and leptin for all CpGs significant at the 5% level in one or both analyses. CpGs significantly associated with both adipokines are shown in red, those significant only in the adiponectin analysis are blue and those only significant in the leptin analysis are coloured yellow. A reference line (*y*=−*x*) is shown by a grey dotted line, indicating perfectly inverse effect sizes between the two adipokines. (**b**) Forest plot of the enrichment of traits in the adipokine-associated CpGs showing log_*e*_-transformed ORs and their 95% CIs. Traits that are in the top ten (as determined by OR) for one or both adipokines are included, and they are ordered by maximum OR in descending order. Results obtained using the adiponectin-associated set of CpGs are shown in blue and those obtained from the leptin analysis are shown in yellow. All enrichments shown are significant at the 5% level after adjusting for multiple testing

The final set of CpGs included 73 adiponectin and 211 leptin-associated sites (Fig. 2), representing 65 and 203 distinct loci, respectively. Ten CpGs were associated with both adipokines, and adiponectin and leptin effect sizes were inversely correlated at the 274 uniquely identified CpGs (*R*=−0.81 *p*<0.001; Fig. 3a).

### Adipokine-associated methylation is also linked to metabolic health

To assess the relevance of the adipokine-associated CpGs, we conducted a search of previous EWAS (ESM Table 5). Notably, 65 of the 73 adiponectin CpGs (89.0%) and 145 of the 211 leptin CpGs (68.7%) were associated with at least one other trait. As anticipated a priori, adipokines and adiposity were closely related and, despite the removal of CpGs sensitive to adjustment for BMI, and therefore evidence for BMI-independent associations with adipokines at the tested CpGs, the strongest enrichments were observed with BMI (OR 571.7 and 151.3 for adiponectin and leptin, respectively; *p*_fdr_<0.001). In total, 40 (54.8%) of the adiponectin and 51 (24.2%) of the leptin CpGs were associated with BMI in large-scale blood-based EWAS. In addition, enrichments existed for other metabolic risk factors (Fig. 3b), including HDL-cholesterol (OR for adiponectin and leptin, respectively, 465.8 and 102.8, *p*_fdr_<0.001), triglycerides (ORs 1917.4 and 413.7, *p*_fdr_<0.001), systolic BP (ORs 215.2 and 137.4, *p*_fdr_<0.001), fasting insulin (ORs 435.5 and 147.5, *p*_fdr_<0.001) and glucose levels (ORs 121.4 and 22.0, *p*_fdr_<0.001), as well as type 2 diabetes itself (ORs 533.8 and 100.6, *p*_fdr_<0.001), highlighting the relevance of our CpGs to metabolic health as a whole.

### Functional genomics uncovers regulatory potential in adipokine CpGs

**Fig. 4:**
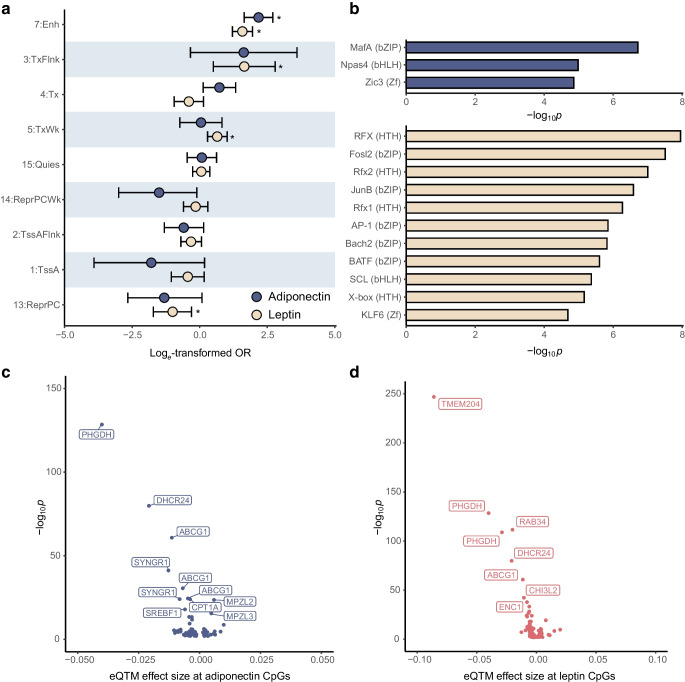
Plots showing regulatory enrichment and integrative analysis results. (**a**) Forest plot of chromatin state enrichments in adiponectin- (blue) and leptin- (yellow) associated CpGs, identified using the PBMC Roadmap reference epigenome (E062). Log_*e*_-transformed ORs and 95% CIs are shown in descending order, with the largest OR at the top. Six states with very large CIs for one or both adipokines are not shown (12_EnhBiv, 11_BivFlnk, 10_TssBiv, 9_Het, 8_ZNF/Rpts and 6_EnhG). *p_fdr_<0.05. Chromatin states shown are referred to in line with the original Roadmap annotation and include enhancers (7:Enh), regions flanking active transcription (3:TxFlnk), transcribed regions (4:Tx), weakly transcribed regions (5:TxWk), quiescent regions (15:Quies), weakly polycomb-repressed regions (14:ReprPCWk), regions flanking active transcription start sites (2:TssAFlnk), active transcription start sites (1:TssA) and polycomb-repressed regions (13:ReprPC). (**b**) Bar plot showing enriched TFBS motifs and −log_10_(*p* value) for adiponectin- (blue) and leptin- (yellow) associated CpGs. Regions within 50 base pairs of the CpG sites were scanned using HOMER and enrichment was quantified by comparing CpG regions with a random genomic background. All TFBS shown are significant at the 5% level. Transcription factors are referred to by their accepted abbreviation, as used within HOMER, and structural motifs are shown within parentheses. (**c**) Volcano plot of the relationships between expression quantitative trait methylation effect sizes and their significance, shown as −log_10_(*p* value). DNAm at the adiponectin-associated CpGs and normalised expression levels of genes within 100 kb of them was investigated in the BIOS consortium. The 11 genes with the highest support are shown. (**d**) Volcano plot of the relationships between expression quantitative trait methylation effect sizes and their significance, shown as −log_10_(*p* value). DNAm at the leptin-associated CpGs and normalised expression levels of genes within 100 kb of them was investigated in the BIOS consortium. The eight genes with the highest support are shown. bHLH, basic helix-loop-helix; bZIP, basic leucine zipper; eQTM, expression quantitative trait methylation; HTH, helix-turn-helix; Zf, zinc finger

We annotated the genomic positions of the 73 adiponectin- and 211 leptin-associated CpGs to 15 chromatin states using Roadmap reference epigenomes [21]. These consist of eight active and seven repressed states showing distinct levels of DNAm, accessibility and regulator binding. By testing if adipokine CpGs were enriched for any particular genomic feature in the PBMC reference (E062), we revealed that active chromatin states were over-represented and repressive states depleted in our results (Fig. 4a and ESM Table 6).

Both adipokines displayed enhancer enrichment, with 18 adiponectin (24.7%, OR8.82) and 33 leptin CpGs (15.6%, OR4.87) annotated to enhancers, compared with only 3.6% of total tested CpGs in the adiponectin (*n*=14,706) and leptin (*n*=14,548) analyses. This indicated high probability of co-localisation with markers of open chromatin, specifically H2K4me1. To investigate whether this pattern was cell-type-specific or tissue-specific, enrichment was analysed using reference epigenomes for 22 other immune cell types (Roadmap Epigenomes: E029–48, E050–51) and adipocytes (E063; ESM Table 7). In all tested epigenomes except one (T_reg_ for adiponectin), CpG genomic locations were enriched for enhancers (*p*_fdr_≤0.05) demonstrating robust regulatory potential independent of cell identity.

Since DNAm influences nearby expression predominantly by modulating TF binding [41], we tested regions within 50 bp of the adipokine-associated CpGs for TFBS enrichments (Fig. 4b and ESM Table 8), revealing links to 14 distinct TFs. Several of these were central to immunity and inflammation (e.g. basic leucine zipper transcription factor, ATF-like [BATF], BTB domain and CNC homologue 2 [Bach2] and activator protein 1 [AP-1]) [42–44], while others had specific adipokine relevance, including Fos-related antigen 2 (Fosl2), which promotes leptin expression [45], and MAF bZIP transcription factor A (MafA), which downregulates adiponectin [46]. Taken together, these functional analyses support adipokine-related DNAm as occurring at *cis-*regulatory regions with potential functional relevance.

### Integrative analyses relate adipokine CpGs to metabolic gene expression

Associations between DNAm and expression of nearby genes (±100 kb) was tested using blood-based data from the BIOS consortium (*n*=3152; ESM Tables 9, 10). Out of 1069 tested CpG–gene pairs, 21.2% were linked in this analysis (*n*=227, *p*_fdr_<0.05), with the majority representing inverse relationships (71.5%) in line with previous reports [47]. Thirty-five (47.9%) adiponectin CpGs were associated with expression of 46 genes (Fig. 4c) and 100 (47.4%) leptin CpGs were linked to 151 genes (Fig. 4d). Of the identified gene–CpG pairs, almost one in six involved the nearest gene in both the adiponectin (15.2%, *n*=7) and leptin (15.9%, *n*=24) analyses. Additionally, DNAm at two distinct CpGs (cg11851174 and cg20544516) was associated with *SREBF1*, a key regulator of lipid homeostasis [48]. In total, there were eight genes overlapping between leptin and adiponectin analyses, several of which are central to lipid transport (e.g. *ABCG1*) [49], biosynthesis (e.g. *DHCR24*) [50] and metabolism (e.g. *CPT1A*) [51].

Biological roles for the implicated genes were interrogated using over-representation analysis. Of 16,037 gene sets tested, 79 were enriched in the 46 genes linked to adiponectin DNAm (ESM Table 11) and 15 were enriched in the 151 leptin genes (ESM Table 12). Findings for both adipokines highlighted links with lipid metabolism (e.g. ‘cholesterol metabolism’ and ‘metabolism of lipids’). Almost half (*n*=7, 46.7%) of the leptin gene sets contained the terms ‘metabolic’ or ‘metabolism’ but this pattern was reduced in the adiponectin-related terms (*n*=10, 12.7%). Adiponectin gene sets were more closely linked to cellular reprogramming, including via AMP-activated protein kinase (AMPK) (*p*_fdr_=3.4 × 10^−3^) and mammalian target of rapamycin complex 1 (mTORC1) (*p*_fdr_=9.7 × 10^−3^) signalling. These pathway-level results highlight the importance of the identified genes in metabolic molecular processes and regulation.

### Bidirectional Mendelian randomisation and triangulation analyses suggest DNAm may drive adiponectin levels

Ascertaining the directionality of relationships in EWAS is not straightforward. To shine light on the most plausible sequence of events, genetic variants can act as proxies for adipokine and DNAm exposures. In line with previous EWAS reporting [5, 52], we performed bidirectional 2SMR followed by triangulation analysis. 2SMR predicts the causal effect of an exposure on an outcome by combining genetically determined levels of both, using GWAS or quantitative trait loci (QTL) databases. Triangulation expands upon these directional inferences and assumes that, if genetically determined outcome levels (‘observed effects’) are driven by the exposure, then they can be predicted by combining genetically determined exposure and exposure–outcome associations (the ‘predicted effect’). The correlation between observed and predicted effects then quantifies the combined support for a causal direction, even if there is insufficient power at the individual CpG level. By performing both analyses bidirectionally, we comparatively inferred which direction of effect is most strongly supported by the data.

**Fig. 5:**
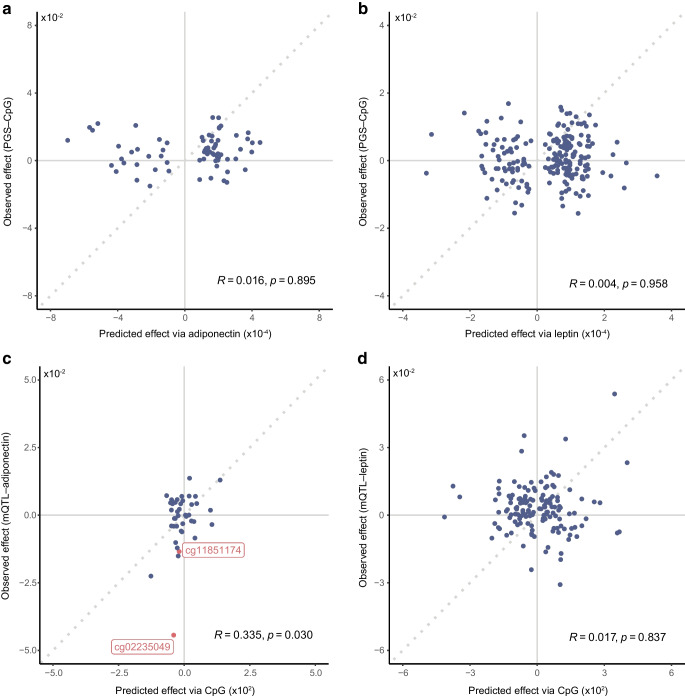
Scatter plots of the results from triangulation analysis showing correlations between predicted (through an exposure) and observed genetic effects on an outcome. Pearson correlation coefficients and their associated *p* values are shown in each plot. (**a**) Predicted (via adiponectin) and observed (PGS–CpG associations) effects for the influence of genetically determined adiponectin on DNAm at adiponectin-associated CpGs. (**b**) Predicted (via leptin) and observed (PGS–CpG associations) effects for the influence of genetically determined leptin on DNAm at the leptin-associated CpGs. (**c**) Predicted (via DNAm) and observed (mQTL–adiponectin) effects for the influence of DNAm at the adiponectin-associated CpGs on serum adiponectin. Two CpGs significant from the 2SMR analysis are shown labelled in red. (**d**) Predicted (via DNAm) and observed (mQTL–leptin) effects for the influence of DNAm at the leptin-associated CpGs on serum leptin. A reference line (*y*=*x*) is shown by a grey dotted line in all plots

In light of the conclusions from previous EWAS, where blood-based DNAm was a consequence rather than a cause of traits [5, 8, 52], we explored whether adipokine levels could be driving DNAm. 2SMR did not suggest that methylation was caused by either adiponectin (ESM Table 13) or leptin (ESM Table 14), and triangulation (ESM Tables 15, 16) consolidated this finding with minimal correlations between observed (PGS–DNAm) and predicted (PGS–adipokine/adipokine–DNAm) effects (*R*<0.02; Fig. 5a, b).

In the reverse direction, 2SMR supported DNAm at two CpGs influencing adiponectin levels (ESM Tables 17, 18). The first, cg11851174 (chr17:17712609), was associated with incident type 2 diabetes [53] and annotated to active chromatin in both PBMCs (4:Tx) and adipocytes (7:Enh). In the blood-based integrative analyses, this site was linked to *SREBF1* (β=−0.004, *p*_fdr_=8.3 × 10^−5^), which encodes a TF central to lipid homeostasis and biosynthesis and whose expression is decreased in obesity and type 2 diabetes [48].

The second CpG putatively driving adiponectin, cg02235049 (chr3:186559186), is a novel site not previously identified in EWAS but strikingly annotated to *ADIPOQ*, which encodes adiponectin. Integrative follow-up into its methylation and nearby *ADIPOQ* expression was hindered in the BIOS consortium data as adiponectin is not produced by immune cells. However, in publicly available DNAm and expression data from SGBS pre-adipocytes (*n*=38), this CpG was negatively correlated with *ADIPOQ* expression (*R*=−0.36, *p*=0.029). This inverse relationship aligned with 2SMR results (β=−0.217, *p*_fdr_=2.1 × 10^−12^) and a functionally repressive effect of DNAm on expression at this adipocyte-specific enhancer.

The 2SMR direction of effect at both CpGs, where DNAm influences adiponectin, was also supported by triangulation analysis (Fig. 5c, d and ESM Tables 19, 20), with observed and predicted effects correlated for adiponectin (*R*=0.335, *p*=0.030) but not for leptin (*R*=0.017, *p*=0.837). Taken together, these findings indicate a cell-type-specific effect for the two CpGs identified as putative drivers of adiponectin, with evidence of links to expression for cg02235049 and cg11851174 in adipocytes and leukocytes, respectively.

### DNAm driving adiponectin is also upstream of metabolic risk and disease

**Fig. 6:**
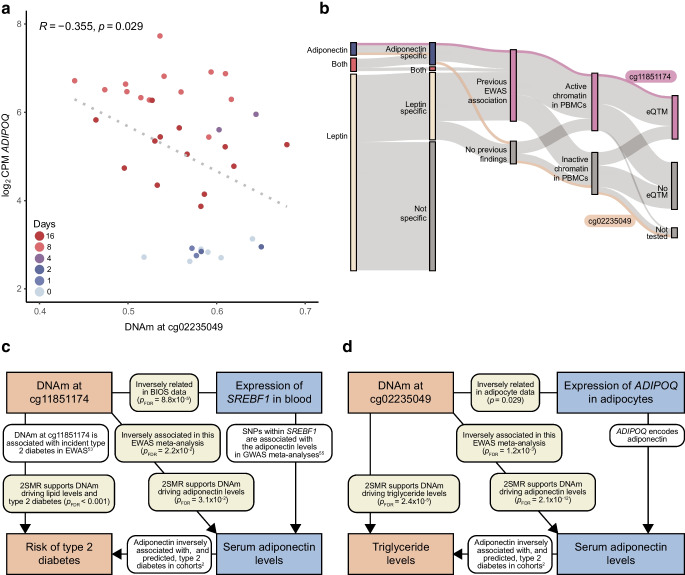
Combined evidence that DNAm at two loci influences metabolic health. (**a**) Scatter plot of the relationship between DNAm at cg02235049 and normalised *ADIPOQ* expression levels in SGBS pre-adipocytes (*n*=38) differentiating over the course of 16 days. Pearson correlation coefficients and their associated *p* values are shown and the line of best fit is shown by a grey dotted line. (**b**) Sankey diagram showing multiple downstream investigations into CpGs significant at the 5% level after adjusting for multiple testing in this adiponectin and/or leptin EWAS meta-analysis. CpGs removed following sensitivity analyses (Not specific) are shown and those kept in due to independent effects with adiponectin, leptin or both continue in the diagram. CpGs not linked to traits in previous EWAS are shown (No previous findings) and those that were linked continue in the diagram. CpGs not lying in regions of active chromatin in PBMCs are shown (Inactive chromatin in PBMCs). Finally, CpGs not linked to expression of genes within 100 kb in the BIOS consortium are shown (No eQTM or Not tested). cg11851174 is shown as having evidence supporting its functional relevance in all of these investigations (highlighted in purple), and cg02235049 is shown as not having such collective evidence (highlighted in orange). (**c**) Flowchart showing the body of evidence supporting DNAm at cg11851174 being linked to type 2 diabetes via *SREBF1* and serum adiponectin: findings from the current study (yellow boxes); increases in traits (DNAm and type 2 diabetes risk; orange boxes); decreased traits (*SREBF1* expression and serum adiponectin; blue boxes); and evidence from previous work (white boxes). All mentioned links are significant at the 5% level after adjustment for multiple testing. (**d**) Flowchart showing the body of evidence supporting DNAm at cg02235049 being linked to triglyceride levels via *ADIPOQ* and serum adiponectin: findings from the current study (yellow boxes); increases in traits (DNAm triglyceride levels; orange boxes); decreased traits (*ADIPOQ* expression and serum adiponectin; blue boxes); and evidence from previous work (white boxes). All mentioned links are significant at the 5% level after adjustment for multiple testing. eQTM, expression quantitative trait methylation

Evidence that these two CpGs (cg11851174 and cg02235049) were more likely a cause than a consequence of adiponectin combined with their links to nearby expression in relevant cell types (*SREBF1* in blood and *ADIPOQ* in adipocytes, respectively; Fig. 6a), prompted deeper analysis of their clinical significance. In particular, for the *SREBF1* CpG (cg11851174) there were multiple lines of evidence pointing towards functional regulation, including previous EWAS, genomic annotation, integrative links and causal inference (Fig. 6b). Using 2SMR, we evaluated causal links between these two CpGs, type 2 diabetes and several metabolic risk factors including fasting insulin and lipid levels.

Methylation of the *SREBF1* CpG (cg11851174) plausibly decreased HDL-cholesterol levels (*p*_fdr_=4.11 × 10^−3^) and increased both fasting insulin (*p*_fdr_=3.84 × 10^−3^) and risk of type 2 diabetes (*p*_fdr_=2.39 × 10^−5^; ESM Table 21). Additionally, both the *SREBF1* and *ADIPOQ* CpGs likely drove triglyceride level increases (*p*_fdr_=2.94 × 10^−2^ and *p*_fdr_=2.39 × 10^−5^ for cg11851174 and cg02235049, respectively). In the reverse direction, there was insufficient evidence for DNAm at either CpG resulting from investigated metabolic traits or type 2 diabetes itself (ESM Table 22). These results showcase these specific loci as upstream epigenetic markers of type 2 diabetes pathogenesis. Coupled with evidence that *SREBF1* expression decreases type 2 diabetes risk by increasing serum adiponectin [54, 55] and the clear relevance of *ADIPOQ* to adiponectin production, there is now considerable support for their direct regulatory potential in metabolic risk (Fig. 6c, d).

## Discussion

In this study, we performed an EWAS of associations between circulating adipokines and genome-wide DNAm in five blood-based cohorts (adiponectin *n*=2791; leptin *n*=3661). Through sensitivity analyses for cell-type proportions, smoking and BMI, we derived two sets of CpGs robustly associated with adiponectin (*n*=73) and leptin (*n*=211). Methylation at these CpGs was associated with both type 2 diabetes and metabolic risk factors including BMI, fasting insulin and HDL-cholesterol. Additionally, integrative analyses linked adipokine-associated DNAm to expression of genes central to transport (e.g. *ABCG1*) [49], biosynthesis (e.g. *DHCR24*) [50] and metabolism (e.g. *CPT1A*) [51] of lipids.

Bidirectional 2SMR and triangulation did not indicate a causal relationship between DNAm and leptin in either direction but did support methylation at two CpGs potentially regulating adiponectin, namely cg02235049 and cg11851174. The first of these is a novel CpG not previously identified in blood-based EWAS. Annotated to *ADIPOQ*, the gene encoding adiponectin, this CpG lies in a repressed chromatin region in PBMCs, making functional relevance for its methylation in leukocytes unlikely. Indeed, *ADIPOQ* was not/lowly expressed in blood according to the BIOS consortium data. However, functional genomics data revealed that this CpG was in an adipocyte-specific enhancer and, since adipocytes are the primary producers of adiponectin, this represented a biologically plausible cell-type-specific effect. Therefore, we investigated the relationship between this CpG and *ADIPOQ* expression in publicly available adipocyte data and observed a significant, inverse correlation that aligned with our causal inference results.

This discovery underscores the potential for genome-wide epigenetic analyses in large-scale blood-based cohorts to identify biologically relevant sites, even where their functional roles may be in less accessible but more metabolically relevant tissues. Such associations could be driven by shared upstream factors, such as diet, causing DNAm in a tissue-agnostic manner. Although these CpGs may only be functional in some tissues (e.g. adipose), their ability to be detected in blood allows well-powered EWAS such as this one to identify biologically meaningful correlations. These sites, and others like them, will ideally serve as focal points for targeted hypothesis-driven investigations into adiponectin production by, for example, experimentally modifying methylation in adipocytes.

The second CpG plausibly driving serum adiponectin levels (cg11851174) resides in active chromatin in both PBMCs and adipocytes close to the *SREBF1* gene, which encodes a TF central to lipid homeostasis that binds to sterol regulatory elements in the promoters of genes including *ADIPOQ* [48, 56]. Therefore, similar to the *ADIPOQ* CpG, this *SREBF1* site could represent a functional epigenetic effect in adipocytes being mirrored in blood. However, since we also linked increased DNAm at this site to decreased *SREBF1* expression in the large-scale blood-based BIOS consortium data, there is another plausible explanation for how DNAm could act upstream of adiponectin production. Previous experimental evidence from macrophage-specific sterol regulatory element-binding protein (SREBP) cleavage-associating protein (SCAP) knockout mice has demonstrated that reduced SREBP-1a activity promotes macrophage polarisation to proinflammatory subtypes [57]. Therefore, circulating monocytes epigenetically primed for lower *SREBF1* expression could feasibly have proinflammatory cell fates as adipose-tissue macrophages. Since local inflammation is an established inhibitor of adiponectin production from adipocytes, this represents a sequence of events where immune cell DNAm could more directly influence adipokine production [58]. These two hypotheses would need to be tested in differentiating monocytes or macrophage–adipocyte co-cultures but our findings offer an indication of plausible mechanisms to follow-up.

Considering the collective evidence at the *ADIPOQ* and *SREBF1* loci, and since the *SREBF1* CpG has also previously been associated with HbA_1c_ [59] and incident type 2 diabetes [53], we investigated the broader implications of these sites for metabolic disease using bidirectional 2SMR. This indicated that these CpGs may also act upstream of metabolic traits, including triglyceride levels and type 2 diabetes. These directional associations, coupled with previous work implicating *SREBF1* in type 2 diabetes risk via adiponectin [54, 55] and the plausible relevance of *ADIPOQ* as the gene encoding adiponectin, reinforce these CpGs as promising epigenetic markers.

There were limitations to our study. Notably, we explored relationships between leukocyte DNAm and serum adiponectin with only minimal follow-up in adipocytes, the cells that predominantly produce adipokines. Future functional experiments in relevant tissues will be needed to test the hypotheses generated here. Additionally, we could not adjust for smoking in our main analysis due to incomplete data and instead opted to ensure smoking-independent effects via a two-step sensitivity analysis restricted to the subset with complete data. While sex was included as a covariate to adjust for potential confounding, no sex-stratified analyses were performed. This limited our ability to determine whether associations differed between sexes. Gender identity was not assessed. Future research could explore whether these findings apply equally across sex and gender groups. This study was also conducted in European populations, and it remains to be tested whether our findings can be generalised to other ethnicities. Lastly, this study was not immune to the common weaknesses of molecular 2SMR. For the adipokine–DNAm 2SMR, data were not available to test all CpGs, meaning that only 57.5% of the adiponectin-associated CpGs and 61.6% of the leptin-associated CpGs were followed up in this analysis, and not all independent SNPs were available in the mQTL and GWAS datasets. This limited, and could have biased, instruments used for these exposures. In addition, most mQTLs with strong effects lie in close proximity to each other and are highly correlated. Only between one and four independent mQTLs existed to instrument each CpG and this 2SMR approach was expected to have limited success in identifying directional effects with bias towards the null [60].

In summary, this study highlights the potential of integrative, epigenome-wide studies to uncover biologically meaningful epigenetic markers of molecular traits, and reveals novel insights into the regulatory mechanisms underlying adiponectin production. By highlighting critical loci, we offer focal points for future experimental research aiming to dissect the secretory profiles of adipocytes or identify therapeutic targets in metabolic disease.

## Supplementary Information

Below is the link to the electronic supplementary material.

ESM Methods (PDF 173 KB)

ESM Tables (XLSX 54.0 MB)

## Data Availability

Summary statistics and other data underlying these findings are available in the ESM. Regarding individual-level data from the cohorts involved, the informed consents given by KORA study participants does not cover data posting in public databases. However, data are available upon request from KORA Project Application Self-Service Tool (https://helmholtz-muenchen.managed-otrs.com/external/Data). Requests for data can be submitted online and are subject to approval by the KORA Board. The data of the SHIP study cannot be made publicly available due to the informed consent of the study participants but it can be accessed through a data application form available at https://transfer.ship-med.uni-greifswald.de/ for researchers who meet the criteria for access to confidential data. The HumanMethylation450 BeadChip data from the LLD and LLS are available as part of the BIOS Consortium in the European Genome-phenome Archive (EGA), under the accession code EGAD00010000887 (https://ega-archive.org/datasets/EGAD00010000887). Additional -omic and phenotype data are available upon request via the BBMRI-NL BIOS consortium. All data can be requested by bona fide researchers from the respective cohorts. Information about the individual studies analysed in this manuscript can be found in ESM Methods. Correspondence and requests for materials should be addressed to the corresponding author. All other data used in this study are publicly available: EWAS summary statistics can be downloaded from the EWAS Catalogue [19] and EWAS atlas [20], PBMC reference epigenome data are available from ROADMAP [21], TFBS data are available within the HOMER software [22], full mQTL summary statistics can be requested from GoDMC [35], adiponectin [36] and leptin [37] GWAS summary statistics are available from the GWAS database, LD proxies and matrices can be accessed using LDlink [38], variances in methylation and expression were calculated from data generated by the BIOS (a full list of investigators is available from https://ega-archive.org/datasets/EGAD00010000887), libraries for GSEA were downloaded directly from Enrichr (https://maayanlab.cloud/Enrichr/), and SGBS adipocyte data are available from GEO (GSE119593 for expression data, GSE119539 for DNAm data). All the software and programs used to conduct these analyses are freely available.

## Abbreviations

2SMR: Two-sample Mendelian randomisation
BIOS: Biobank-based Integrative Omics Studies
CPM: Counts per million
DMP: Differentially methylated position
DMR: Differentially methylated region
DNAm: DNA methylation
EA: Effect allele
EAF: Effect allele frequency
EWAS: Epigenome-wide association study
FDR: False discovery rate
GEO: Gene Expression Omnibus
GoDMC: Genetics of DNA Methylation Consortium
GWAS: Genome-wide association study
IV: Instrumental variable
IVW: Inverse variance weighted
KORA: Cooperative Health Research in the Region of Augsburg
LD: Linkage disequilibrium
LLD: LifeLines DEEP
LLS: Leiden Longevity Study
mQTL: Methylation quantitative trait locus
NK: Natural killer
PBMC: Peripheral blood mononuclear cell
PGS: Polygenic score
RIN: Rank inverse normal
SGBS: Simpson-Golabi-Behmel syndrome
SHIP: Study of Health in Pomerania
SREBP: Sterol regulatory element-binding protein
TF: Transcription factor
TFBS: Transcription factor binding site

## Code availability

Custom code for the respective analyses is available at https://github.com/nebulyra/adipo_ewas.
