# Supplementary material for: DNA methylation of genes involved in lipid metabolism drives adiponectin levels and metabolic disease
Source: Diabetologia. 2025 Oct 8;69(1):127–45. doi: 10.1007/s00125-025-06549-6 (PMC12686031; doi:10.1007/s00125-025-06549-6)
Supplement: Supplementary file 1 — ESM Methods (PDF 173 KB) [file 125_2025_6549_MOESM1_ESM.pdf]

Electronic Supplementary Material (ESM) Methods

Cohort Descriptions

Leiden Longevity Study (LLS)

The LLS [1] is a cohort of long-lived, Dutch, Caucasian siblings (n=944), who were recruited with their offspring (n=1,671) and their offspring's partners (n=744). Between 2002 and 2006, research nurses collected non-fasted blood samples from living study participants for isolation of DNA, RNA, serum, and plasma, and these were stored at -80˚C until analysis. From these samples, adiponectin levels were determined with the DuoSet ELISA kit (R&D Systems Europe Ltd, Abingdon, United Kingdom) according to the manufacturer's instructions. The lower and upper detection limits of the assay were respectively 62.5 and 4000 pg/ml. Serum leptin was measured using 'two-step' sandwich ELISA kit (Diagnostics Biochem Canada Inc., Dorchester, Ontario, Canada) according to manufacturer's instructions. Assay sensitivity was 0.5 ng/ml.

DNA methylation data of whole blood samples was generated from 821 unrelated participants from the offspring-partner generation of the LLS by the Human Genotyping facility (HuGe-F, Erasmus MC, Rotterdam, The Netherlands) within the Biobank-Based Integrative Omics Studies (BIOS) consortium, funded by BBMRI-NL, a research infrastructure financed by the Dutch government (NOW 184.021.007). Genomic DNA (500ng) was bisulfite converted using the Zymo EZ-96 DNA methylation kit (Zymo Research Corp, Irvine, CA, USA). 4 µl was then hybridized on the Infinium HumanMethylation450 BeadChip array (Illumina Inc, San Diego, CA, USA) according to the manufacturer's protocol. Data preprocessing and quality control followed the DNAmArray workflow [2].

Written informed consent for DNA collection and its use for genetic analyses was obtained from all participants prior to their enrolment into the study. Good clinical practice guidelines

were maintained, and the study protocol was approved by the local Medical Ethical Committee of the Leiden University Medical Center.

Kooperative Gesundheitsforschung in der Region Augsburg (KORA F4)

The KORA F4 study (2006-2008) [3] followed up individuals (n=3,080) living in the region of Augsburg, Southern Germany, aged between 32 and 81 years old. Blood samples were drawn after an 8 hour fast and frozen at -80˚C until analysis. Serum leptin concentrations were determined using ELISAs from Mecodia, Stockholm, Sweden. The intra- and inter-assay coefficients of variation for leptin were <10%. Serum adiponectin levels were measured in the subsample aged 61-82 years using the Quantikine ELISA kit from R&D Systems (Wiesbaden, Germany) [4]. The intra- and inter- assay coefficients of variation were 3.8% and 8.0%, respectively.

Following isolation according to standard procedures, genomic DNA (750 ng) from 1,707 whole blood samples was bisulfite converted with the Zymo EZ-96 DNA Methylation Kit (Zymo Research Corp, Irvine, CA, USA). 4 µl from each sample underwent amplification, enzymatic fragmentation, and application to the Infinium HumanMethylation450 BeadChip array (Illumina Inc, San Diego, CA, USA) according to the manufacturer's protocol [5]. GenomeStudio with the methylation module was used to extract and process the raw image data and initial quality assessment was conducted using the Control Dashboard. For data pre-processing, a common pipeline [6] was used.

In accordance with the Declaration of Helsinki, written informed consent was obtained from all participants prior to their enrolment into the study. Good clinical practice guidelines were maintained, and the study protocol was approved by the Ethics Committee of the Bavarian Medical Association.

TwinsUK

Fasting morning serum total adiponectin levels were measured with a two-site DELFIA assay using antibodies and standards from R&D Systems (Minneapolis, MN). The day-to-day coefficient of variation (CV) for adiponectin was 9.9% at a concentration of 3.2 ng/ml, 7.8% at 8.6 ng/ml, and 5.2% at 14.7 ng/ml. Serum leptin concentration was determined after an overnight fast using an RIA (Linco Research, St. Louis, MO).

As described previously [7], fasting whole blood DNAm of individuals in TwinsUK was profiled using the Infinium HumanMethylation450 BeadChip (Illumina Inc, San Diego, CA). DNAm was assessed at over 450 thousand sites and processing of methylation signals was performed with R Bioconductor software [8]. Briefly, the ENmix package [9] was used for quality control of the data, and the minfi package [10] was used to exclude samples with median methylated and unmethylated signal ratio less than 10.5. Background correction, dye bias correction, and quantile normalization were performed with ENmix as described previously [11]. Underperforming probes and outlier samples were identified using standard parameter values and signals with detection *p*-value above 1E-6 and number of beads fewer than three were excluded from the analysis. Maximum probe and sample missingness were set to 5%.

The Study of Health in Pomerania – TREND (SHIP-TREND)

The Study of Health in Pomerania – Trend (SHIP-TREND) is a longitudinal population-based cohort study in West Pomerania, a region in the northeast of Germany, assessing the prevalence and incidence of common population-relevant diseases and their risk factors. Baseline examinations for SHIP-TREND were carried out between 2008 and 2012, comprising 4,420 participants aged 20 to 81 years. Study design and sampling methods were previously described [12].

DNA was extracted from blood samples (n=508) of SHIP-TREND participants to assess DNA methylation using the Illumina HumanMethylationEPIC BeadChip array. Samples available for

this project were randomly selected based on the availability of multiple OMICS data, and excluding type II diabetes. The samples were taken between 07:00 AM and 04:00 PM, and serum aliquots were prepared for immediate analysis and for storage at -80 °C in the Integrated Research Biobank (Liconic, Liechtenstein). Processing of the DNA samples was performed at the Helmholtz Zentrum München. Preparation and normalization of the array data was performed according to the CPACOR workflow [13] using the software package R. The array IDAT files were processed using the minfi package [10]. Probes that had a detection *p*-value above background (sum of per-array methylated and unmethylated intensity values-based p-value ≥1E-16) were set to missing. Methylation beta values were calculated as proportion of methylated intensity value on the sum of methylated + unmethylated + 100 intensities. Arrays with observed technical problems (±4SD outside control probe intensity mean) during steps like bisulfite conversion, hybridization or extension, as well as arrays with mismatch between sex of the proband and sex determined by the chr X and Y probe intensities were removed from subsequent analyses. Additionally, only arrays with a call rate ≥ 95% were processed further resulting in 495 samples with methylation data on 865,859 sites available, and 441 samples with phenotype data available for subsequent analyses. In addition to sex, age, and white blood cell type counts, the first six principal components of the control probe intensities obtained by the CPACOR workflow were included in the model as covariates to account for technical factors. Details on assessment of the metabolic phenotypes and covariates used in this analysis are provided within the SHIP cohort design publication [12].

The medical ethics committee of the University of Greifswald approved the study protocol, and oral and written informed consents were obtained from each of the study participants.

LifeLines DEEP (LLD)

A total of 701 adults from the Lifelines DEEP cohort were included in this study based on available epigenome-wide methylation data and cytokines. Initially, 1539 participants were enrolled in the Lifelines DEEP, which is a subpopulation of the Lifelines cohort in the north of The Netherlands [14, 15]. Except for the regular Lifelines procedures, additional deep molecular measurements were performed in Lifelines DEEP participants. The plasma levels of leptin (ng/ml) and adiponectin (μg/ml) were measured by enzyme-linked immunosorbent assay (ELISA) platform.

For genome-wide DNA methylation data, 500 ng of genomic DNA was bisulfite-converted using the EZ DNA Methylation kit (Zymo Research Corp., USA) and hybridized on Illumina HumanMethylation450 BeadChip arrays (Illumina, Inc.) according to the manufacturer's protocols. The original IDAT files were generated by the Illumina iScan BeadChip scanner and performed by the Human Genotyping facility (HugeF) of ErasmusMC, The Netherlands (http://www.glimDNA.org/). Quality control and normalization details are described elsewhere [16]. Methylation levels at each CpG site were expressed as the ratios of the methylated intensity over the total intensity (β-values), which were used for the subsequent statistical analyses.

All participants provided written informed consent. The Lifelines DEEP study was approved by the Medical Ethical Committee of the University Medical Center Groningen (UMCG), Groningen, The Netherlands.

References

1. Schoenmaker M, de Craen AJM, de Meijer PHEM, et al (2006) Evidence of genetic enrichment for exceptional survival using a family approach: The Leiden Longevity Study. European Journal of Human Genetics 14(1):79–84. https://doi.org/10.1038/SJ.EJHG.5201508,
2. Sinke L, van Iterson M, Cats D, Slieker R, Heijmans B DNAmArray: Streamlined workflow for the quality control, normalization, and analysis of Illumina methylation array data. https://doi.org/10.5281/ZENODO.3355292
3. Meisinger C, Strassburger K, Heier M, et al (2010) Prevalence of undiagnosed diabetes and impaired glucose regulation in 35-59-year-old individuals in Southern Germany: The KORA F4 study. Diabetic Medicine 27(3):360–362. https://doi.org/10.1111/J.1464-5491.2009.02905.X,
4. Herder C, Bongaerts BWC, Rathmann W, et al (2013) Association of subclinical inflammation with polyneuropathy in the older population: KORA F4 study. Diabetes Care 36(11):3663–3670. https://doi.org/10.2337/DC13-0382,
5. Kriebel J, Herder C, Rathmann W, et al (2016) Association between DNA Methylation in whole blood and measures of glucose metabolism: Kora F4 study. PLoS One 11(3). https://doi.org/10.1371/JOURNAL.PONE.0152314,
6. Touleimat N, Tost J (2012) Complete pipeline for Infinium® Human Methylation 450K BeadChip data processing using subset quantile normalization for accurate DNA methylation estimation. Epigenomics 4(3):325–341. https://doi.org/10.2217/EPI.12.21,
7. Costeira R, Evangelista L, Wilson R, et al (2023) Metabolomic biomarkers of habitual B vitamin intakes unveil novel differentially methylated positions in the human epigenome. Clin Epigenetics 15(1):166. https://doi.org/10.1186/S13148-023-01578-7
8. Gentleman RC, Carey VJ, Bates DM, et al (2004) Bioconductor: open software development for computational biology and bioinformatics. Genome Biol 5(10):R80. https://doi.org/10.1186/GB-2004-5-10-R80
9. Xu Z, Niu L, Li L, Taylor JA (2016) ENmix: A novel background correction method for Illumina HumanMethylation450 BeadChip. Nucleic Acids Res 44(3). https://doi.org/10.1093/NAR/GKV907,
10. Aryee MJ, Jaffe AE, Corrada-Bravo H, et al (2014) Minfi: A flexible and comprehensive Bioconductor package for the analysis of Infinium DNA methylation microarrays. Bioinformatics 30(10):1363–1369. https://doi.org/10.1093/BIOINFORMATICS/BTU049,
11. Christiansen C, Tomlinson M, Eliot M, et al (2022) Adipose methylome integrative-omic analyses reveal genetic and dietary metabolic health drivers and insulin resistance classifiers. Genome Med 14(1). https://doi.org/10.1186/S13073-022-01077-Z,
12. Völzke H, Schössow J, Schmidt CO, et al (2022) Cohort Profile Update: The Study of Health in Pomerania (SHIP). Int J Epidemiol 51(6):E372–E383. https://doi.org/10.1093/IJE/DYAC034,
13. Lehne B, Drong AW, Loh M, et al (2015) A coherent approach for analysis of the Illumina HumanMethylation450 BeadChip improves data quality and performance in

epigenome-wide association studies. Genome Biol 16(1). https://doi.org/10.1186/S13059-015-0600-X,
14. Scholtens S, Smidt N, Swertz MA, et al (2015) Cohort Profile: LifeLines, a three-generation cohort study and biobank. Int J Epidemiol 44(4):1172–1180. https://doi.org/10.1093/IJE/DYU229,
15. Tigchelaar EF, Zhernakova A, Dekens JAM, et al (2015) Cohort profile: LifeLines DEEP, a prospective, general population cohort study in the northern Netherlands: study design and baseline characteristics. BMJ Open 5(8):e006772. https://doi.org/10.1136/BMJOPEN-2014-006772
16. Lu X, Fraszczyk E, van der Meer TP, et al (2020) An epigenome-wide association study identifies multiple DNA methylation markers of exposure to endocrine disruptors. Environ Int 144. https://doi.org/10.1016/j.envint.2020.106016
